# Physiology of Hyperuricemia and Urate-Lowering Treatments

**DOI:** 10.3389/fmed.2018.00160

**Published:** 2018-05-31

**Authors:** Caroline L. Benn, Pinky Dua, Rachel Gurrell, Peter Loudon, Andrew Pike, R. Ian Storer, Ciara Vangjeli

**Affiliations:** ^1^Pfizer Ltd., Cambridge, United Kingdom; ^2^DMPK, Oncology, IMED Biotech Unit, AstraZeneca, Cambridge, United Kingdom; ^3^IMED Biotech Unit, Medicinal Chemistry, Discovery Sciences, AstraZeneca, Cambridge, United Kingdom

**Keywords:** URAT1, xanthine oxidase, crystal deposition, uric acid, kidney disease, hypertension, diabetes

## Abstract

Gout is the most common form of inflammatory arthritis and is a multifactorial disease typically characterized by hyperuricemia and monosodium urate crystal deposition predominantly in, but not limited to, the joints and the urinary tract. The prevalence of gout and hyperuricemia has increased in developed countries over the past two decades and research into the area has become progressively more active. We review the current field of knowledge with emphasis on active areas of hyperuricemia research including the underlying physiology, genetics and epidemiology, with a focus on studies which suggest association of hyperuricemia with common comorbidities including cardiovascular disease, renal insufficiency, metabolic syndrome and diabetes. Finally, we discuss current therapies and emerging drug discovery efforts aimed at delivering an optimized clinical treatment strategy.

## Introduction

Hyperuricemia is a condition characterized by abnormally elevated levels of serum urate (sUA), while gout, the most common form of inflammatory arthritis, arises from the subsequent deposition of urate crystals when concentrations become saturated. Gout has been defined as “a progressive metabolic disease characterized by symptomatic hyperuricemia and deposition of monosodium urate (MSU) crystals in joints and soft tissues due to an imbalance in uric acid uptake, synthesis or excretion” (cited in (1)). The initial clinical sign of an acute gout attack is severe disabling pain, usually involving a single joint, which typically spontaneously resolves over a period of a few days to weeks without intervention, although treatment with anti-inflammatory drugs such as colchicine, NSAIDs (non-steroidal anti-inflammatory drugs) and corticoids will generally improve symptoms more rapidly. Upon resolution of an acute attack the patient will enter a symptom-free interval, however flares can recur with increased frequency and duration if the underlying pathology is not addressed. If sUA values remain high, MSU crystal deposits can grow and expand to other sites leading to further inflammation and associated tissue/joint injury. Ultimately a subset of individuals will transition to chronic tophaceous gout which is characterized by nodular urate crystal deposits, recurrent flares and concurrent arthritis, which takes 11.6 years on average to occur from the initial flare (reviewed in (2)).

It is often stated that the prevalence of hyperuricemia and gout has increased in recent years, although there are relatively few longitudinal studies in geographically diverse populations and increasing diagnosis rates may play a significant role. It has been highlighted that the distribution of gout varies significantly across the world which may reflect factors such as ethnicity, diet and socioeconomic factors (3). In the US the National Health and Nutrition Examination Survey [NHANES] 2007–2008 suggested estimating prevalence of gout hyperuricemia and gout at approximately 21 and 4%, respectively, an increase of 3.2 and 1.2% respectively when compared to the prior NHANES-III study conducted from 1988 to 1994 (4, 5). Likewise, a review of data collected in the Australian population suggested an increase in the prevalence of gout from 0.5 to 1.7% from 1968 to 1995/6 (6). However, this trend is not universal and data collected in Taiwan over a similar time period, 1993–1996 and 2005–2008, to the US study showed a fall in the prevalence of hyperuricemia from 25.3 to 22.0% in men and from 16.7 to 9.7% in women (7). Some authors have argued that this is related to the epidemic of obesity and associated dietary shift toward foods rich in purines, alcohol consumption and fructose-sweetened drinks (8, 9); however, this is still disputed and indeed, the impact of dietary intervention may be limited with respect to management of sUA (10).

Many epidemiological studies have shown that hyperuricemia and gout are associated with the development of hypertension, cardiovascular disease, chronic kidney disease and diabetes, potentially through crystal-independent modes of action (11–16). Interpretation of these studies is confounded by the specific definition of hyperuricemia that is applied, which in turn contributes to the controversy around the notion of urate having a causal role in these conditions (17). However, it is notable that EULAR (European League Against Rheumatism) now recommends that gout could be seen as a red flag for associated cardiovascular risk factors and co-morbidity, and that blood pressure, lipids and glucose be checked and treated if abnormal (18).

In this article, we review the current knowledge of the physiological and genetic factors involved of uric acid handling in humans. Furthermore, we discuss literature data which examine if hyperuricemia may be a factor in certain comorbidities such as hypertension, cardiovascular disease and chronic kidney disease. Finally, we highlight several current and future therapeutic options for the treatment of hyperuricemia and gout.

## Uric acid

**Figure 1:**
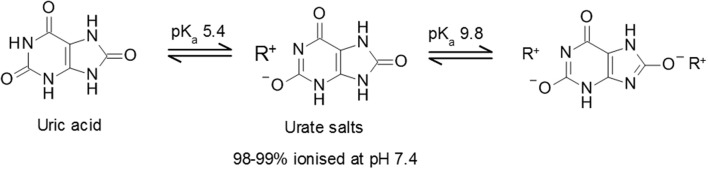
Uric acid pKa and formation of urate salts. Structures of uric acid and urate salts including acid dissociation constants.

Uric acid is a weak diprotic acid with an aqueous pK_a1_ of 5.4 and pK_a2_ of 9.8 (19) (Figure 1). Consequently, at physiological pH, uric acid is predominantly (98–99%) found as the deprotonated urate anion. The solubility of uric acid at normal physiological pH is generally given as 6.8 mg/dL while the reference ranges for sUA are 3.5 to 7.2 mg/dL (210–430 μmol/L) and 2.6–6.0 mg/dL (155–360 μmol/L) in males and premenopausal females respectively (20). It is notable that the upper limit of the normal male reference range includes concentrations that exceed the concentration at which uric acid precipitates.

One interesting aspect of urate biology is the observation that the normal range for serum urate concentrations in humans, and some other primates, are significantly above the typical mammalian range of 0.5–2.0 mg/dL (30–120 μmol/L). The evolutionary drive for acquisition of comparatively high levels of circulating urate remains unclear, although it has been proposed to confer evolutionary advantages as an anti-oxidant, particularly in the context of neurodegenerative diseases such as Parkinson's (21, 22). However, clinical data are conflicting and approaches seeking to increase urate levels have not shown a positive impact on disease outcomes (23, 24). In contrast, while any potential benefits of high sUA levels remain to be elucidated it has been clearly demonstrated that hyperuricemia, in conjunction with genetic and/or environmental factors, can lead to significant health problems associated with urate crystal deposition.

### Sources of uric acid

Uric acid is produced during the metabolism of both endogenous (daily synthesis rates approximate 300–400 mg) and exogenous (dietary contribution, approximately 300 mg) purines within a total pool size of 1,200 mg in healthy males (600 mg in females) on a purine-free diet (25, 26). The relationship between diet and sUA is likely to be more complex than simple purine intake, for example beer and sweetened soft drinks (with high fructose corn syrup as a particularly maligned source) have both been shown to have impact on sUA levels independently of their purine content (9, 27, 28). Indeed, consistent with the notion of a direct relationship between fructose intake and high sUA levels, it has been shown that increased fructokinase activation leads to the rapid generation of uric acid which in turn upregulates fructokinase expression (29, 30).

**Figure 2:**
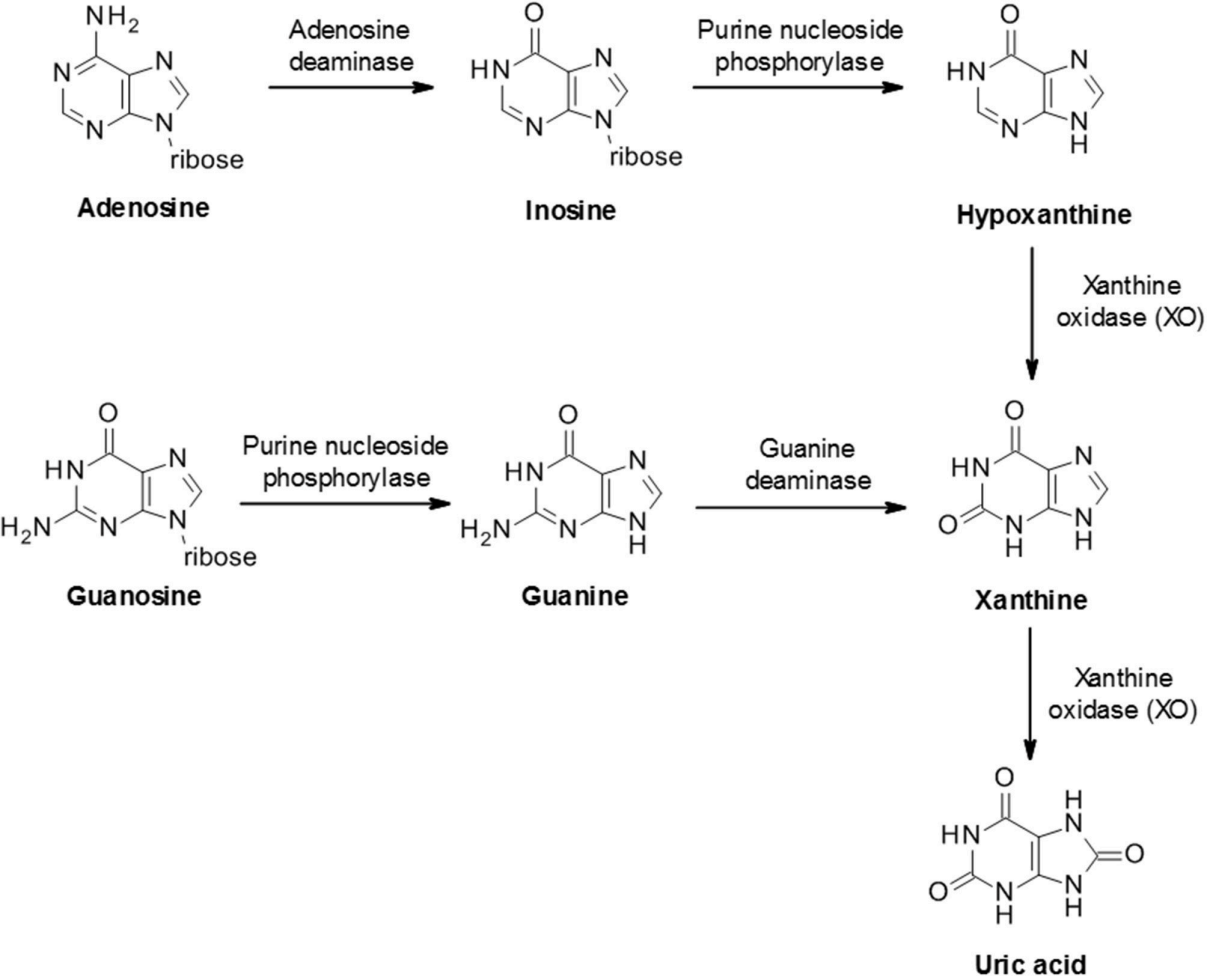
Biosynthesis of uric acid from purines. Purine mononucleotides are catabolized to produce uric acid although the underlying pathway can vary in different tissues and cells. A schematic example pathway is shown.

The biosynthesis of uric acid is catalyzed by the enzyme xanthine oxidase (XO, also known as xanthine oxidoreductase or XOR), coded for by the xanthine dehydrogenase gene *XDH* (31). The enzyme is normally present as an inactive NAD-dependent cytosolic dehydrogenase precursor, which is subsequently subjected to further processing by oxidation or proteolytic modification to form active enzyme. Xanthine oxidase is widely distributed throughout various organs including the liver, gut, lung, kidney, heart, and brain as well as the plasma and is involved in two stages of uric acid generation; conversion of hypoxanthine to xanthine and subsequently xanthine to uric acid (Figure 2).

### Uric acid clearance

**Figure 3:**
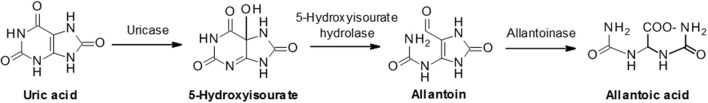
Uric acid metabolism via uricase. In humans and some primates, uric acid is the final product of the purine catabolism pathway. However, most animals further degrade uric acid to allantoic acid via the sequential actions of uricase, 5-hydroxyisourate hydrolase and allantoinase.

In most mammalian species uric acid is further metabolized by the enzyme uricase to the more soluble allantoin (Figure 3) which is subsequently excreted in the urine. However, humans and some higher order primates lack a functional uricase enzyme and therefore uric acid is the final breakdown product of the pathway (21). This discrepancy in uric acid handling between species can represent a significant challenge in the preclinical evaluation of urate lowering drugs during drug discovery.

Urate elimination from humans occurs via two main routes; approximately two-thirds being excreted in urine with normal uricosuria levels of 620 ± 75 mg/day in adult, while the remainder is thought to be largely excreted via the gastrointestinal tract (25, 26, 32, 33). Hyperuricemia may also be associated with hyperuricosuria (defined as urinary excretion of urate >800 mg/day in men and >750 mg/day in women). Urate elimination can be quantified as clearance (normal males: 8.7 ± 2.5 mL/min) or as fractional excretion of urate (FEUA) which indicates the net urate excretion by the kidney (normal males: 7.25 ± 2.98%). Healthy subjects have an average FEUA in the range of 6–8%, whereas gout patients generally have average FEUA of 3–5% (34–36). These observations are consistent with the notion that decreased renal excretion or low FEUA represents a major contributor to hyperuricemia as opposed to increased generation of uric acid.

**Figure 4:**
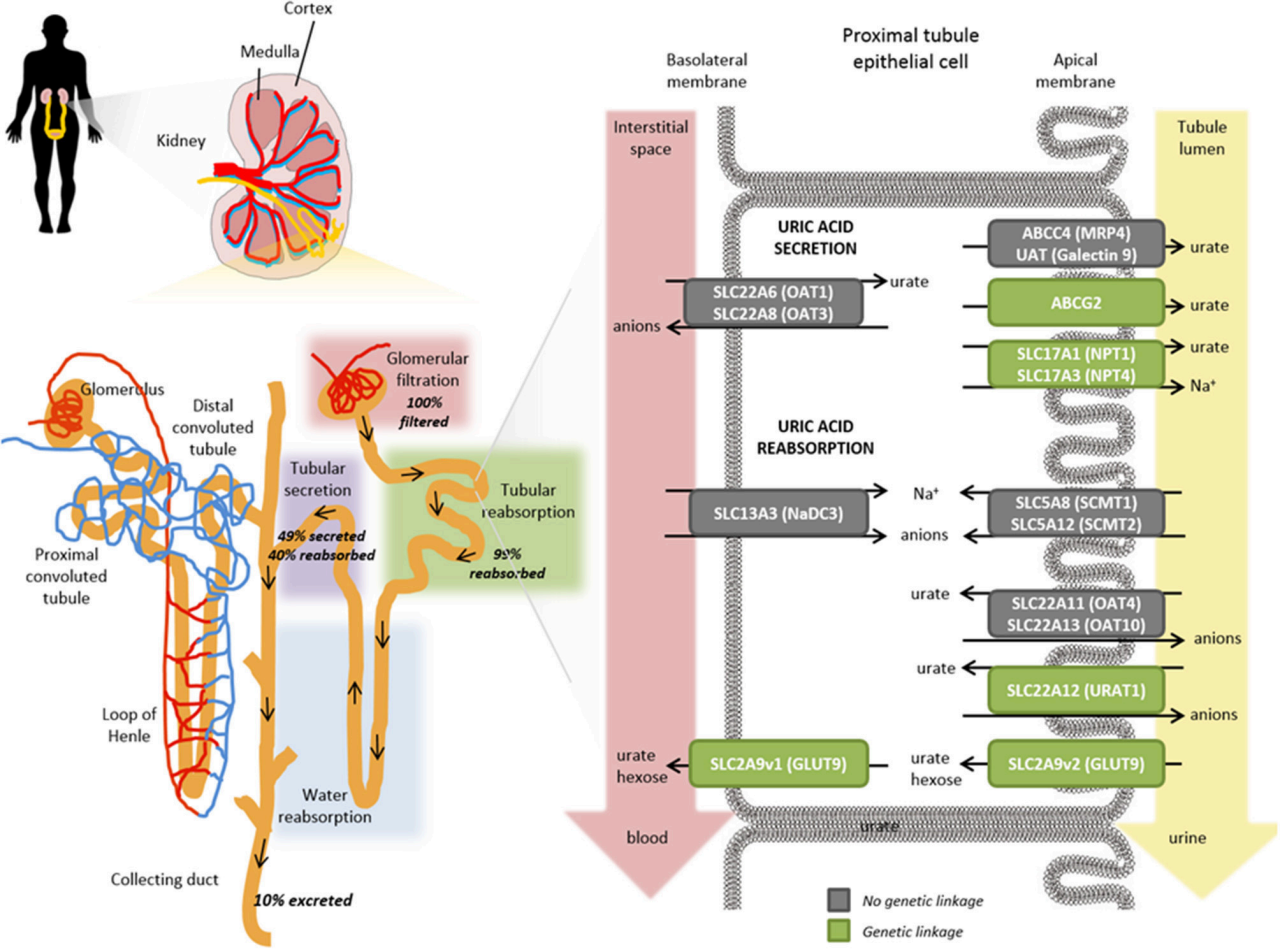
Role of transporters in the renal proximal tubule on urate handling. Within an individual nephron in the kidney (yellow), filtration of water and solutes occurs in the glomerular capsule from the afferent arteriole into the renal tubule (pink shading). Tubular reabsorption (green shading) is predominantly mediated by the proximal convoluted tubule whereas tubular secretion extracts uric acid (and other substances) from peritubular capillaries (purple shading) and secretes them into the tubular fluid for urinary excretion. Urate transporters in renal proximal tubule epithelial cells actively mediate the secretion and reabsorption of urate. The balance between these processes determines the net excretion levels from the kidney. The anion transporters SLC22A6 (OAT1) and SLC22A8 (OAT3) localized on the basolateral membranes transport urate from the interstitial space in the blood depending on the gradients for exchanged anions but have not been shown to exhibit a genetic linkage with hyperuricemia or gout risk (gray box). On the apical membrane, ABCG2, SLC17A1 (NPT1), SLC17A3 (NPT4), ABCC4 (MRP4), UAT (Galectin 9) have all been shown to contribute to the secretory transport of urate into the tubule lumen and leading to urinary excretion; a number of these have been genetically associated with hyperuricemia and gout risk (green boxes). Exchange gradients upstream of urate anion exchange are enabled through the actions of SLC13A3 (NaD3), SLC5A8 (SCMT1), and SLC5A12 (SCMT2). In renal reabsorption, the apical urate-anion exchanger SLC22A12 (URAT1) has been shown to play a predominant role in urate homeostasis and indeed several variants have been identified to be associated with gout and hyperuricemia risk (green box). Additional contributions to urate reabsorption are mediated by SLC22A11 (OAT4) and SLC22A11 (OAT10) (gray boxes, not genetically associated with gout/hyperuricemia risk) and the short isoform of SLC2A9v2 (GLUT9, green box) on the apical membrane. The long isoform of SLC2A9v1 (GLUT9, green box) is the only known transporter to mediate basolateral efflux of urate back into circulation; which is in accordance with its genetic association for gout and hyperuricemia risk in addition to rare mutations associated with hypouricemia.

Despite the high fraction of renally excreted uric acid, the process is more complex than simple glomerular filtration, with approximately 91–95% of filtered urate being reabsorbed in the proximal tubule. Reabsorption is a key factor underpinning the comparatively high levels of circulating urate and is primarily mediated by transporters that exchange intracellular anions for urate (37, 38). Reabsorption and secretion of urate predominates in the S1 and S2 regions of the proximal tubule although it is not clear whether the secretion happens concomitantly with reabsorption and/or if there is post-reabsorptive secretion within the tubule. Ultimately, around 3–10% of the filtered urate emerges in the urine. Several transporters playing a role in reabsorption and secretion have been identified (Figure 4).

### Urate reabsorption transporters

#### URAT1 *(SLC22A12)*

The identification of URAT1 (*SLC22A12)* as the dominant apical (luminal) urate exchanger in the human proximal tubule was a landmark event in the understanding of urate homeostasis (39). URAT1 is a 12-transmembrane domain protein, predominantly expressed on the apical brush border membrane of proximal tubule epithelial cells in the kidneys. URAT1 has been shown to transport urate with a K_m_ of 371 ± 28 μM as well as other organic anions such as orotate, salicylate, lactate and nicotinate (39–41). URAT1-mediated urate transport is a tertiary active process dependent on sodium gradients which are initially established via basolateral Na^+^K^+^ATPases which actuate a number of apical Na^+^ coupled organic anion transporters, in turn providing the driving force for urate reabsorption (42, 43).

Clinical genetic studies have confirmed that loss-of-function mutations of URAT1 are associated with FEUA of 40–100% and extremely low serum urate levels (average levels of 0.93 mg/dL) (44) (Table 1). Interestingly, it has been shown that testosterone increases and estradiol decreases URAT1 protein levels in mice and it is interesting to speculate whether this would contribute to the increased hyperuricemia susceptibility in males and post-menopausal women (89, 90). Additional genetic susceptibility toward hyperuricemia and gout via *PDZK1* association is potentially through its known function of modulating the apical membrane localization of URAT1 (57, 91) (Table 1). While URAT1 appears to be the predominant apical reabsorption transporter, other pathways are inferred given that FEUA is <100% even in patients with complete URAT1 loss of function mutations.

**Table 1:** Genetic associations with urate levels.

**Genomic location**	Candidate gene^*^	**Protein encoded by candidate gene**	**Function (if known) provided by RefSeq**	**Genetic association with gout/hyperuricemia**	**References**
4q22.1	*ABCG2*	BCRP	The membrane-associated protein encoded by this gene is included in the superfamily of ATP-binding cassette (ABC) transporters which transport various molecules across extra- and intra-cellular membranes. ABCG2 can function as a xenobiotic transporter which may play a major role in multi-drug resistance. It likely serves as a cellular defense mechanism in response to mitoxantrone and anthracycline exposure	Multiple common variant associations with serum urate levels, renal under-excretion gout and overall risk of gout	(45–56)
2q22.3	*ACVR2A*	Activin A receptor type 2	This gene encodes a transmembrane serine-threonine kinase receptor that mediates the functions of activins (members of the transforming growth factor-beta (TGF-beta) superfamily). This gene may be associated with susceptibility to preeclampsia, a pregnancy-related disease which can result in maternal and fetal morbidity and mortality	Common variant association with serum urate levels	(50)
10q11.2	*ASAH2*	N-acylsphingosine amidohydrolase 2	Ceramidases (EC 3.5.1.23) such as ASAH2, catalyze hydrolysis of the N-acyl linkage of ceramide, a second messenger in a variety of cellular events, to produce sphingosine. Sphingosine exerts both mitogenic and apoptosis-inducing activities, and its phosphorylated form functions as an intra- and intercellular second messenger	Common variant association with serum urate levels	(50)
17q23.2	*C17ORF82*	Chromosome 17 open reading frame 82	Unknown	Common variant association with serum urate levels	(50)
2p23.3	*GCKR*	Glucokinase regulator	The gene product is a regulatory protein that inhibits glucokinase in liver and pancreatic islet cells by binding non-covalently to form an inactive complex with the enzyme. This gene is considered a susceptibility gene candidate for a form of maturity-onset diabetes of the young (MODY)	Common variant association with serum urate levels	(50)
17q22	*HLF*	HLF, PAR bZIP transcription factor	The encoded protein forms homodimers or heterodimers with other PAR family members and binds sequence-specific promoter elements to activate transcription. Chromosomal translocations fusing portions of this gene with the E2A gene cause a subset of childhood B-lineage acute lymphoid leukemias	Common variant association with serum urate levels	(50)
8q21.13	*HNF4G*	Hepatocyte nuclear factor 4 gamma	This gene is also known as NR2A2 (nuclear receptor subfamily 2, group A, member 2). HNF4 was originally classified as an orphan receptor that exhibits constitutive transactivation activity through fatty acid bindings. Mutations in the HNF4A gene have been linked to maturity onset diabetes of the young 1 (MODY1)	Common variant association with serum urate levels	(50)
15q26.3	*IGF1R*	Insulin like growth factor 1 receptor	This receptor binds insulin-like growth factor with a high affinity. It has tyrosine kinase activity. Cleavage of the precursor generates alpha and beta subunits. The insulin-like growth factor I receptor plays a critical role in transformation events and is highly overexpressed in most malignant tissues where it functions as an anti-apoptotic agent by enhancing cell survival	Common variant association with serum urate levels	(50)
2q14.2	*INHBB*	Inhibin beta B subunit	This gene encodes a member of the TGF-beta (transforming growth factor-beta) superfamily of proteins. The encoded preproprotein is proteolytically processed to generate a subunit of the dimeric activin and inhibin protein complexes. Polymorphisms near this gene are associated with pre-eclampsia in female human patients	Common variant association with serum urate levels	(50)
12q13.3	*INHBE*	Inhibin beta E subunit	This gene encodes a member of the TGF-beta (transforming growth factor-beta) superfamily of proteins. The encoded preproprotein is proteolytically processed to generate an inhibin beta subunit. This gene may be upregulated under conditions of endoplasmic reticulum stress, and this protein may inhibit cellular proliferation and growth in pancreas and liver	Common variant association with serum urate levels	(50)
2q31.1	*LRP2*	LDL receptor related protein 2	The protein encoded by this gene is a multi-ligand endocytic receptor that is expressed in many different tissues but primarily in absorptive epithelial tissues such as the kidney. The LRP2 protein is critical for the reuptake of numerous ligands, including lipoproteins, sterols, vitamin-binding proteins, and hormones. This protein also has a role in cell-signaling. Mutations in this gene cause Donnai-Barrow syndrome (DBS) and facio-oculoacoustico-renal syndrome (FOAR)	Common variant association with serum urate levels	(46)
11q13.1	*LTBP3*	Latent transforming growth factor beta binding protein 3	The protein encoded by this gene forms a complex with transforming growth factor beta (TGF-beta) proteins and may be involved in their subcellular localization. This protein also may play a structural role in the extracellular matrix	Common variant association with serum urate levels	(50)
16q23.2	*MAF*	MAF bZIP transcription factor	The protein encoded by this gene is a DNA-binding, leucine zipper-containing transcription factor that acts as a homodimer or as a heterodimer. Defects in this gene are a cause of juvenile-onset pulverulent cataract as well as congenital cerulean cataract 4 (CCA4)	Common variant association with serum urate levels	(50)
7q11.23	*MLXIPL*	MLX interacting protein like	This gene encodes a basic helix-loop-helix leucine zipper transcription factor of the Myc/Max/Mad superfamily. This protein forms a heterodimeric complex and binds and activates, in a glucose-dependent manner, carbohydrate response element (ChoRE) motifs in the promoters of triglyceride synthesis genes. The gene is deleted in Williams-Beuren syndrome, a multisystem developmental disorder caused by the deletion of contiguous genes at chromosome 7q11.23	Common variant association with serum urate levels	(50)
3p21.1	*MUSTN1*	Musculoskeletal, embryonic nuclear protein 1	Unknown	Common variant association with serum urate levels	(50)
16q22.1	*NFAT5*	Nuclear factor of activated T-cells 5	Proteins belonging to this family play a central role in inducible gene transcription during the immune response. This protein regulates gene expression induced by osmotic stress in mammalian cells	Common variant association with serum urate levels	(50)
15q24.2	*NRG4*	Neuregulin 4	The neuregulins, including NRG4, activate type-1 growth factor receptors to initiate cell-to-cell signaling through tyrosine phosphorylation	Common variant association with serum urate levels	(50)
1q21.1	*PDZK1*	PDZ domain containing 1	PDZ domain-containing molecules bind to and mediate the subcellular localization of target proteins. Single nucleotide polymorphisms in this gene may be associated with metabolic syndrome, and overexpression of this gene may play a role in drug resistance of multiple myeloma	Common variant association with serum urate levels	(50, 57)
1q22	*PKLR*	Pyruvate kinase, liver and RBC	The protein encoded by this gene is a pyruvate kinase that catalyzes the transphosphorylation of phohsphoenolpyruvate into pyruvate and ATP, which is the rate-limiting step of glycolysis. Defects in this enzyme, due to gene mutations or genetic variations, are the common cause of chronic hereditary nonspherocytic hemolytic anemia (CNSHA or HNSHA)	Common variant association with serum urate levels	(50)
7q36.1	*PRKAG2*	Protein kinase AMP-activated non-catalytic subunit gamma 2	This gene is a member of the AMPK gamma subunit family. AMPK is an important energy-sensing enzyme that monitors cellular energy status and functions by inactivating key enzymes involved in regulating de novo biosynthesis of fatty acid and cholesterol. Mutations in this gene have been associated with Wolff-Parkinson-White syndrome, familial hypertrophic cardiomyopathy, and glycogen storage disease of the heart	Common variant association with serum urate levels	(50)
17q25.1	*PRPSAP1*	Phosphoribosyl pyrophosphate synthetase associated protein 1	Unknown	Common variant association with serum urate levels	(50)
12q24.13	*PTPN11*	Protein tyrosine phosphatase, non-receptor type 11	The protein encoded by this gene is a member of the protein tyrosine phosphatase (PTP) family which regulate a variety of cellular processes including cell growth, differentiation, mitotic cycle, and oncogenic transformation. Mutations in this gene are a cause of Noonan syndrome as well as acute myeloid leukemia	Common variant association with serum urate levels	(50)
6p24.3	*RREB1*	Ras responsive element binding protein 1	The protein encoded by this gene is a zinc finger transcription factor that binds to RAS-responsive elements (RREs) of gene promoters	Common variant association with serum urate levels	(50)
4p16.1	*SLC2A9*	GLUT9	This gene encodes a member of the SLC2A facilitative glucose transporter family. Members of this family play a significant role in maintaining glucose homeostasis. The encoded protein may play a role in the development and survival of chondrocytes in cartilage matrices	Multiple common variant associations with serum uric acid levels, renal overload gout, renal under-excretion gout and overall risk of gout. Low frequency variants associated with renal hypouricemia 2	(45–51, 54–56, 58–77)
10q21.2	*SLC16A9*	Solute carrier family 16 member 9	Unknown	Common variant association with serum urate levels	(50)
6p22.2	*SLC17A1*	NPT1	Sodium-dependent phosphate transport protein 1 is a protein encoded by the SLC17A1 gene	Multiple common variant associations with serum uric acid levels and gout risk	(45, 48, 50, 53, 56, 78–80)
6p22.2	*SLC17A3*	NPT4	The protein encoded by this gene is a voltage-driven transporter that excretes intracellular urate and organic anions from the blood into renal tubule cells. The longer isoform is a plasma membrane protein with transporter activity while the shorter isoform localizes to the endoplasmic reticulum	Multiple common variant associations with serum uric acid levels. Rare loss-of-function variants found in patients with hyperuricemia	(50, 53, 81)
11q13.1	*SLC22A11*	OAT4	The protein encoded by this gene is involved in the sodium-independent transport and excretion of organic anions. OAT4 is an integral membrane protein and is found mainly in the kidney and in the placenta	Multiple common variant associations with serum uric acid levels	(45, 47, 50, 54)
11q13.1	*SLC22A12*	URAT1	The protein encoded by this gene is a member of the organic anion transporter (OAT) family, and it acts as a urate transporter to regulate urate levels in blood. This protein is an integral membrane protein primarily found in epithelial cells of the proximal tubule of the kidney. An elevated level of serum urate, hyperuricemia, is associated with increased incidences of gout, and mutations in this gene cause renal hypouricemia type 1	Multiple common variant associations with serum uric acid levels, renal overload gout, renal under-excretion gout and overall risk of gout. Low frequency variants associated with renal hypouricemia type I	(39, 44–46, 50, 54, 56, 82–85)
8p21.2	*STC1*	Stanniocalcin 1	This gene encodes a secreted, homodimeric glycoprotein that is expressed in a wide variety of tissues and may have autocrine or paracrine functions. The protein may play a role in the regulation of renal and intestinal calcium and phosphate transport, cell metabolism, or cellular calcium/phosphate homeostasis. Overexpression of human stanniocalcin 1 in mice produces high serum phosphate levels, dwarfism, and increased metabolic rate. This gene has altered expression in hepatocellular, ovarian, and breast cancers	Common variant association with serum urate levels	(50)
5q13.2	*TMEM171*	Transmembrane protein 171	Unknown	Common variant association with serum urate levels	(50)
6p21.1	*VEGFA*	Vascular endothelial growth factor A	This growth factor induces proliferation and migration of vascular endothelial cells, and is essential for both physiological and pathological angiogenesis. Elevated levels of this protein are found in patients with POEMS syndrome, also known as Crow-Fukase syndrome. Allelic variants of this gene have been associated with microvascular complications of diabetes 1 (MVCD1) and atherosclerosis	Common variant association with serum urate levels	(50)
2p23.1	*XDH*	Xanthine oxidase	Xanthine dehydrogenase belongs to the group of molybdenum-containing hydroxylases involved in the oxidative metabolism of purines. Defects in xanthine dehydrogenase cause xanthinuria, may contribute to adult respiratory stress syndrome, and may potentiate influenza infection through an oxygen metabolite-dependent mechanism	Rare loss-of-function variants found in patients with Type I xanthinuria	(86, 87)

* *For most loci discovered through GWAS, the causal gene is yet to be determined. For loci reported in Kottgen et al. (50), the candidate gene listed here is based on the GRAIL prediction (88). For all others, the candidate gene is that in which the lead associated variant resides*.

URAT1 is now a well-established drug target with a number of primary and secondary uricosurics (drugs capable of increasing FEUA) such as benzbromarone, probenecid and lesinurad, known to derive at least part of their efficacy through this mechanism albeit, in earlier cases, this was not understood when they were developed (92–97). Interestingly, compounds such as pyrazinamide have been shown to trans-stimulate URAT1 activity to impact on vectorial transport of urate (72, 98).

#### GLUT9 *(SLC2A9)*

Variants in glucose transporter 9 (GLUT9, also referred to as URATv1), coded for by the *SLC2A9* gene, are strongly associated with both hyperuricemia and gout, a finding that has been successfully replicated in multiple studies (59, 99) (Table 1). In addition, individuals with homozygous mutations in GLUT9 present with pronounced hypouricemia and hyperuricosuria with FEUA's of 100% or greater (indicative of net active urate secretion), response to fructose load, and a propensity for nephrolithiasis and exercise induced renal failure (62, 84, 100–102).

GLUT9 appears to function predominantly as a facilitative urate uniporter with at least some additional capacity for hexose transport (59, 103, 104). GLUT9-mediated urate transport is voltage dependent with currents recorded at physiological pH, but independent of sodium, chloride and other ions. Consequently, GLUT9 is distinct from other members of the glucose transporter (SLC2) family due to its substrate specificity and sequence identity although it shares common structural features such as 12 transmembrane helices, cytoplasmic termini, and an N-linked glycosylation site. Two isoforms of GLUT9 have been described that differ only by the first 29 residues of the N-terminal domains (104). The short isoform (GLUT9a) appears to be expressed at both apical and basolateral membranes in proximal tubule epithelium cells (and indeed may contribute to the import of urate from the peritubular interstitium and thus facilitate renal urate secretion). The long isoform (GLUT9b) is predominantly expressed on the basolateral membrane and is the only known basolateral efflux transporter for urate.

Interestingly, a positive relationship has been described between glycosuria and uricosuria suggesting that there could be interference between the tubular reabsorption of glucose and the tubular capacity to reabsorb urate (105). Indeed, it has been proposed that SGLT2 inhibitor treatment lowers serum uric acid through alteration of uric acid transport activity in renal tubules. This is consistent with clinical observations with canagliflozin treatment decreasing serum urate in patients with type 2 diabetes (a known co-morbidity of hyperuricemia), including those with baseline hyperuricemia (106–108). Therefore, studies which assess urate lowering therapy (ULT) efficacy should also consider ambient glycemia which could contribute to the uricosuric effect (109).

#### OAT4 (*SLC22A11*) and OAT10 (*SLC22A13*)

Organic anion transporters 4 (OAT4) and 10 (OAT10) have a range of organic anion substrates and are expressed on the apical membrane of proximal tubule epithelium cells together with URAT1 (*SLC22A12*) (99). While both transporters have been demonstrated to exhibit low levels of urate transport capabilities, only OAT4 has been associated with hyperuricemia and gout together with inefficient renal secretion (78,80).

### Urate excretion transporters

#### ABCG2/BCRP (*ABCG2*)

Genetic variation in human ABCG2, an ATP-driven efflux pump on the apical membrane proximal tubule epithelial cells, has emerged as a major factor in human hyperuricemia and gout risk (78, 110, 111). Of note, the landmark paper from Ichida and colleagues shows that decreased extra-renal urate excretion caused by ABCG2 dysfunction contributes to clinical hyperuricemia; although paradoxically, urinary urate excretion is also increased by ABCG2 dysfunction implying further roles for regulation of urate levels by this transporter (110). However, ABCG2 is also expressed in the intestine and thus a contribution of gastrointestinal transport cannot be ruled out (110, 112).

#### NPT1 (*SLC17A1*) and NPT4 (*SLC17A3*)

GWAS (Genome Wide Association Study) studies have shown that NPT1 and NPT4 are associated with hyperuricemia and gout (38, 55, 79, 99). Both NPT1 and NPT4 can transport urate *in vitro* and are localized on the apical membrane supporting the notion of a role in renal secretion of urate from the apical membrane (81, 113, 114). However, their relative importance to urate transport in proximal tubule epithelial cells remains incompletely defined.

#### OAT1 (*SLC22A6*) and OAT3 (*SLC22A8*)

The basolateral entry of urate into renal proximal tubule cells is driven, at least partially, by the outwardly directed gradient for dicarboxylates, which in turn is generated by Na^+^-dependent uptake. Thus, urate exchange is significantly trans-stimulated by dicarboxylates (115). This is further complicated by the observation that OAT1 and OAT3 appear to exchange urate with divalent anions including dicarboxylates, suggesting that they are suited to basolateral entry of urate (38, 116). However, there is no supporting genetic data for a role for these transporters in hyperuricemia.

### Genetic variation influencing uric acid handling

As indicated in the former sections on urate resorption and secretion transporters, much is now known about genetic variants contributing to sUA levels and gout through the discovery of rare monogenic disorders affecting uric acid homeostasis, from GWAS, and from candidate gene studies of common variants at the population level. Indeed, there is a strong overlap across genes implicated in monogenic uricemic traits and gout, in a range of genes in addition to those coding for uric acid transporters (Table 1). The largest GWAS to date, by the Global Urate Genetics Consortium (GUGC), studied >140,000 subjects of European ancestry and found 28 genetic loci associated with sUA levels and gout (50). The per-allele effect sizes on sUA ranged from 0.035 to 0.379 mg/dL. For gout, each allele contributed small effects on disease risk ranging from a 3% decrease to a 73% increase (50). The effects of common genetic variants found to be associated with hyperuricemia and gout in Europeans were of a similar magnitude when tested for association with subjects of other ancestries including African, Indian and Japanese (50). While GWAS have been pivotal in the identification of loci associated with sUA and gout risk, such studies do not provide a direct link to causal genes. For some loci there are obvious and highly likely causal genes, while at other loci the causal gene is far from clear and so GWAS findings should be interpreted with this in mind (117). In addition, genetics is a likely contributor to the well-documented observation that certain ethnic groups have a higher risk than others for hyperuricemia and gout (118, 119). Consistent with this notion, GWAS of distinct ethnic groups has revealed some novel loci for gout and uric acid levels (46, 48, 49). However, in this context, it is worth emphasizing that while hyperuricemia is the central risk factor for gout development, there are other variables in play including age, dietary factors and medications which could be impactful across ethnicities. The heritability of sUA concentrations has been estimated at 42–73% (120, 121), while early twin studies have led to estimates for the heritability of renal urate clearance at 60% and of fractional excretion of urate to be 87% (122). Further family studies have found a significant sUA correlation between siblings, parents and offspring though segregation analysis suggests this is likely due to multiple genetic factors rather than a major Mendelian gene (123).

Hypouricemia is defined when a serum urate concentration is less than or equal 2.0 mg/dL and is reported to occur in 0.8% of hospitalized patients and 0.2% of the general population (124). However, it is possible that the prevalence of hypouricemia is actually higher but undiagnosed (125). Hypouricemia can be associated with decreased fractional excretion of uric acid and increased xanthine excretion (e.g., hereditary xanthuria caused by an autosomal recessive deficiency of *XDH*; (126). More typically, hypouricemia is associated with high fractional excretion of uric acid due to genetic causes; including mutations in genes such as *SLC22A12* (URAT1) (39, 127) and *SLC2A9* (GLUT9) (62, 84) or factors such as uricosuric usage, renal tubulopathy, neoplasias, and other conditions (124, 127). The majority of individuals with hypouricemia are asymptomatic which suggests that it is potentially safe to lower sUA levels to <1 mg/dL although clinical management aims at maintaining sUA levels of 4–6 mg/dL. However, nephrolithiasis and exercise induced renal failure (EIRF) have been reported in individuals with mutations in either the *SLC22A12* (URAT1) or *SLC2A9* (GLUT9) but not *XDH* (XO) genes (82, 128, 129). The underlying mechanism is unclear and hypotheses include: (1) acute urate nephropathy caused by increased urate production during exercise, culminating in its intratubular precipitation; (2) ischaemic renal hypoperfusion secondary to vasoconstriction of the renal vessels, mediated by the production of oxygen free radicals during exercise or; (3) the accumulation of anions not eliminated in patients with URAT1 or GLUT9 gene mutations exerts a toxic tubular effect leading to acute tubular necrosis.

## Hyperuricemia and gout

Hyperuricemia is commonly defined as a serum urate concentration >6.8 mg/dL, based on the *in vivo* solubility of urate above which crystal deposition may occur leading to gout. However, it should be noted that alternative definitions of hyperuricemia are sometimes applied, a factor which needs to be considered when attempting comparative analysis of published data (17). The aqueous solubility of uric acid (6.8 mg/dL) is relatively low when compared to normal range of serum concentrations, and therefore a modest increase can elevate the risk of monosodium urate (MSU) crystal formation and precipitation, notably in the joints and urine reviewed in Chhana et al. (130).

Not all individuals with hyperuricemia go on to develop gout and therefore gout represents a subset of individuals with symptomatic hyperuricemia (131). Although sustained hyperuricemia is a prerequisite of crystal formation, it is not possible to accurately predict which individuals will go on to develop gout, even for those with very high sUA (reviewed in (132)). There is no global consensus on approaches for asymptomatic hyperuricemia nor in response to acute gout attacks, with different strategies advocated by national or international guidelines (133), which range from reactive approaches to active sUA management such as that employed by the Japanese (18, 134–136).

The process of crystal deposition leading to gout is reversible by means of reducing sUA levels below its saturation point and it can be surmised that the rate of crystal reduction will be modulated by both the total crystal load and reduction in sUA (reviewed in (137)). While rapid dissolution of crystal deposits may be desirable in therapeutic sense, urate lowering therapy (ULT) initiation generally leads to increased flare rate and associated pain, potentially as a direct consequence of urate crystal dissolution leading to the removal of a protein deposit protecting the underlying surface from attack by inflammatory cells (reviewed in (138)). Indeed, the increased flare rate when initiating ULT has been hypothesized to underpin the reported low levels of patient compliance (139, 140) highlighting the need for appropriate prophylaxis in the first few months of the initiation phase (141, 142); a consideration reflected in EULAR guidelines which recommend prophylaxis for the first 6 months of therapy (18). It is worth noting that treatment with anti-inflammatories alone may modulate the acute inflammatory response to crystals but it is unlikely to alter crystal deposition and ongoing joint damage. As a result, the patient may be unaware of progressive tophi formation and destruction of cartilage and bone. Better understanding of the extent of crystal deposition is being obtained with the advent of advanced imaging modalities including MRI, ultrasound and CT (143). MSU crystals are found in the synovial fluid in 12.5–90% of gout patients during otherwise asymptomatic phases, suggestive of ongoing inflammation and damage (144). Clinical observations confirm that dissolution of crystals by appropriate sUA lowering treatment results in reduction and ultimately elimination of chronic inflammation (145, 146). Indeed, recently updated EULAR recommendations (18) now advocate active management of urate levels from the first presentation of acute gouty flares at <6 mg/dL (or <5 mg/dL in those with chronic, tophaceous gout) together with improved patient education, including the importance of compliance with long-term treatment, and appropriate pain prophylaxis.

Uric acid urolithiasis refers to the development of a stone or calculus composed of significant amounts of urate in the renal pelvis, ureter, or bladder and are reported to account for 5.0–16.5% of all kidney stones (reviewed in (147–149)). Uric acid crystals may initiate calcium oxalate precipitation by the induction of heterogeneous nucleation. The glomerular filtrate of blood is usually acidified by the kidneys from a pH of approximately 7.4 to approximately 6.0 in the urine although this may vary from 4.5 to 8.0 depending on the individual. Accordingly, uric acid solubility is modulated as a function of its weakly acidic pK_a_ and hence, individuals with more acidic urine (pH 5.5) are more likely to have increased uric acid stone formation than individuals with a normal pH range (pH 6.0–6.5) (reviewed in (147)). Urinary alkalinisztion should therefore reduce stone growth/recurrence, and promote stone dissolution (reviewed in (2)). Up to 20% of patients with gout develop kidney stones although stone formation may also occur in patients with normal urinary and serum levels of urate (reviewed in (147)). MSU crystal deposits have been detected in the renal medulla of patients with gout and furthermore renal function has been shown to be improved after successful urate-lowering treatment in gout patients (reviewed in (150)). This implies that the presence of MSU crystals in the kidneys and the associated inflammation may contribute to renal insufficiency. Hence, urate crystal elimination may contribute to improved renal function and reduce kidney injury or disease.

## Epidemiology of hyperuricemia and associated comorbidities

In addition to the previously mentioned increase in the prevalence of hyperuricemia and gout, cross-sectional case-control studies have found hyperuricemia to be comorbid with multiple common conditions, including cardiovascular events such as coronary artery disease and hypertension (151–153), chronic kidney disease (152, 154) or type 2 diabetes (reviewed in (155, 156)). However, it is unknown whether hyperuricemia is a causal factor in the development of these conditions, is driven by the same risk factors, or if it is a consequence of the manifestation of these disorders. Unless it is directly causal, uric acid lowering is unlikely to be a successful strategy for the treatment of these co-morbid diseases.

If the genetic determinants of hyperuricemia are also associated with the risk of a disease, this provides evidence for causality for hyperuricemia for that disease. This reasoning underpins Mendelian randomization whereby naturally occurring genetic variants are used as instrumental variables to estimate the causal effect on a trait. As many of the common genetic determinants of uric acid levels have been revealed through GWAS, these instrumental variables have been used in several studies to test for the causality of uric acid in multiple disorders including sudden cardiac death (157), blood pressure (158), coronary heart disease (159), chronic kidney disease progression (102), type 2 diabetes (160, 161), triglyceride levels (162) or adiposity (163). However, it remains a challenge to identify the underlying causal mechanisms behind any such associations given the large number of these instruments, with each contributing just a small effect on uric acid levels, across multiple genes. An additional consideration is that many patients with hyperuricemia and gout who develop major cardiovascular and renal events also possess several other known traditional risk factors which may be potentially confounding.

A recent review of the evidence for causality of hyperuricemia in disease concluded that the only robust evidence to date, based on both randomized clinical trials and/or Mendelian randomization, is that of a causal role for hyperuricemia in gout and nephrolithiasis (164). A limitation of the Mendelian randomization approach however is that the genetic instruments for serum urate levels may not adequately capture any effect of intracellular urate levels on disease so this should be considered when interpreting any negative findings (165). Trials seeking to determine whether urate-lowering therapy may influence outcomes in other diseases have been performed in small cohorts for short durations (166, 167); we would like to see replication in larger cohorts with longitudinal assessment.

### Cardiovascular disease

Over the last decade, we have seen an accumulating body of evidence which implicates gout and/or uric acid elevation as an independent predictor for hypertension, atrial fibrillation and cardiovascular disease (150, 155, 168–171). However, clearly not all patients with hyperuricemia go on to develop cardiovascular disease and many of those who do also exhibit one or more other established risk factors.

Clinical observations showing an association between high uric acid levels and hypertension are further corroborated in the pediatric and adolescent populations where blood pressure values are significantly elevated (>95th percentile) in the presence of sUA levels of >5.5 mg/dL (172–174). Furthermore, independently of the pharmacological mechanism (both XO inhibitor and uricosuric), reduction of sUA has been shown to lower blood pressure in early primary hypertension in adolescents (173, 175). This finding is supported by the observation that the nonsynonymous variant in *SLC2A9*, rs16890979 (Val253Ile) is significantly associated both with a reduction in both uric acid and in blood pressure in an Amish cohort exposed to sodium-controlled diets (158). Finally, a number of studies have confirmed that people with elevated sUA are at risk of having high blood pressure, even if they otherwise appear to be perfectly healthy (152, 176) and reviewed in Richette et al. (150).

Both experimental and clinical evidence suggests that deleterious effects of high uric acid levels on cardiovascular disease may occur at the vascular level. For example, it has been shown that high levels of uric acid are associated with low-grade inflammatory state and vascular activation of the renin-angiotensin system (reviewed in (155)). Clinical data further supports the notion of endothelial dysfunction associated with sUA elevation and chronic inflammation (177) and reviewed in Richette et al. (150). An increasing number of researchers have suggested that XO plays an important role in various forms of ischemic and other types of tissue and vascular injuries, inflammatory diseases, and chronic heart failure (reviewed in (178)). Interestingly, common variants in the XDH gene are associated with blood pressure and hypertension although these findings need to be further substantiated (179). Accordingly, XO inhibitors may have more profound effects through restoration of endothelial function as opposed to a lowering of sUA levels *per se*. However the studies were limited in size and careful meta-analyses may be warranted (reviewed in (170)). This question of whether XO inhibitors have potential therapeutic benefit in cardiovascular disease, has been the subject of a number of clinical investigations (175, 180–183). However, the impact of early intervention in patients with asymptomatic hyperuricemia (with or without crystal deposition) on vascular outcomes has not been definitively demonstrated (184). Given the long history of this class of drugs, in particular allopurinol, it is interesting to speculate on the potential value of mining medical record databases to understand the implications of long term urate lowering, although interpretation of such data may be undermined by lack of insight into patient adherence to therapy and by suboptimal dose management (185–187). Mendelian randomization studies have produced mixed results—in subjects from the Ludwigshafen Risk and Cardiovascular Health Study, a higher burden of variants associated with uric acid levels was associated both with an increased risk of cardiovascular death and sudden cardiac death (157). Meanwhile, White et al demonstrated that the evidence for causality of urate levels in coronary artery disease is dependent on the model used. Using the Egger method for Mendelian randomization, which accounts for unmeasured pleiotropy of the instrumental variables, they find that the evidence for causality using traditional Mendelian randomization approaches may be inflated (159). Finally, by excluding instrumental variants shown to be pleiotropic, Keenan et al found a lack of significant association between 14 urate-specific variants and risk of coronary heart disease, ischemic stroke and heart failure (188).

### Kidney disease

It is well accepted that hyperuricemia is associated with crystal-related pathologies such as nephrolithiasis. High plasma urate levels are associated with an increased risk of acute kidney injury (AKI) (154, 189, 190). Studies further suggest potential for renal injury in a manner analogous to cardiovascular outcomes due to renal vasoconstriction via inflammation, endothelial dysfunction and renin-antiotensin system activation (reviewed in (191, 192)). Interestingly, sUA reduction has been found to improve renal function in patients irrespective of gout presentation and a more comprehensive investigation of reno-protective potential for urate lowering therapies may be warranted (193–199). A small Mendelian randomization study supports the hypothesis that hyperuricemia, driven by an instrumental variant in *SLC2A9*, is causal in chronic kidney disease progression (102). Contrary to expectations, a study using a genetic risk score composed of five instrumental variants in uric acid transporters was significantly associated with better (rather than worse) renal function (200). The authors speculate however that it is the activity of these uric acid transporters, rather than the serum urate levels, that are having the protective effect on renal function and that using fractional excretion of uric acid as the exposure in a Mendelian randomization study, instead of serum urate, would help to determine if this is the case.

Clinical data suggests that most gout sufferers under-excrete uric acid leading to a corresponding increase in sUA levels (78, 110, 201). The kidneys play a major role in the regulation of serum uric acid levels given the extensive handling of urate by the renal proximal tubules (as discussed in the physiology section); which adds to the debate whether hyperuricemia merely acts as an indicator of renal dysfunction or has a causative role. Accordingly, there is a wealth of clinical data which supports the hypothesis that hyperuricemia is both a predictor of onset and a modulator of progression for both acute kidney injury and for chronic kidney disease (CKD), the latter of which in particular is increasingly recognized as a global health problem (13, 16, 202–207). In contrast, other epidemiological studies have reported no significant relationship between hyperuricemia and CKD progression (208) and therefore the debate continues.

### Type 2 diabetes

In population-based studies, hyperuricemia was shown to be an independent risk factor for developing Type 2 diabetes (T2D) (60, 209). In cardiovascular disease, there is a complex interplay of factors with obesity, insulin resistance and diuretic use all being associated with increased urate reabsorption confounding efforts to determine the relationship between sUA levels, kidney function and diabetes, in addition to other common co-morbidities or complications such as peripheral neuropathy (210–211). Consistent with the notion of addressing hyperuricemia to impact on symptoms, there are studies suggesting ULT beneficially modulates diabetic-associated phenotypes (194, 213–216). Despite this, Mendelian randomization studies conclude that uric acid is not causal in type 2 diabetes (161, 188), though the accompanying commentary for the former again highlights some of the limitations of Mendelian randomization using instruments that are context specific which may not represent what is happening at the physiological level (109).

The development of sodium glucose co-transporter 2 (SGLT2) inhibitors for the treatment of diabetes has rekindled interest and debate into the role of sUA in diabetes and CKD. SGLT2 inhibitors act via the kidneys in an insulin-independent manner to improve glycemic control but also reduce sUA levels without obvious uric acid nephrolithiasis (106, 216, 217). In one small study, it was shown that SGLT2 inhibitor treatment contributed to decreased plasma UA levels together with increased FEUA levels (109). It is not clear how SGLT2 inhibition impacts on uricosuria although one possibility could be through effects on GLUT9 via induction of glycosuria which in turn would contribute to higher levels of urate exchange across the apical membrane of tubular cells into the urine (105, 106, 218).

Taken together, these studies indicate that hyperuricemia is commonly observed in patients together with co-morbidities such as hypertension, cardiovascular disease, CKD and T2D. To date there are no conclusive data that modulation of uric acid levels reduces that risk (17). Therefore, there is no consensus on therapeutic approaches for these conditions in the context of hyperuricemia and indeed ULT are not indicated for these conditions. Should further studies support the notion that chronic hyperuricemia is associated with increased risk of these debilitating co-morbidities, and furthermore if active reduction of sUA levels proves to be protective, we wonder if this will enable a shift in clinical practice toward proactively monitoring of uric acid levels at least in the first instance.

## Launched (marketed and withdrawn) drugs for hyperuricemia

**Figure 5:**
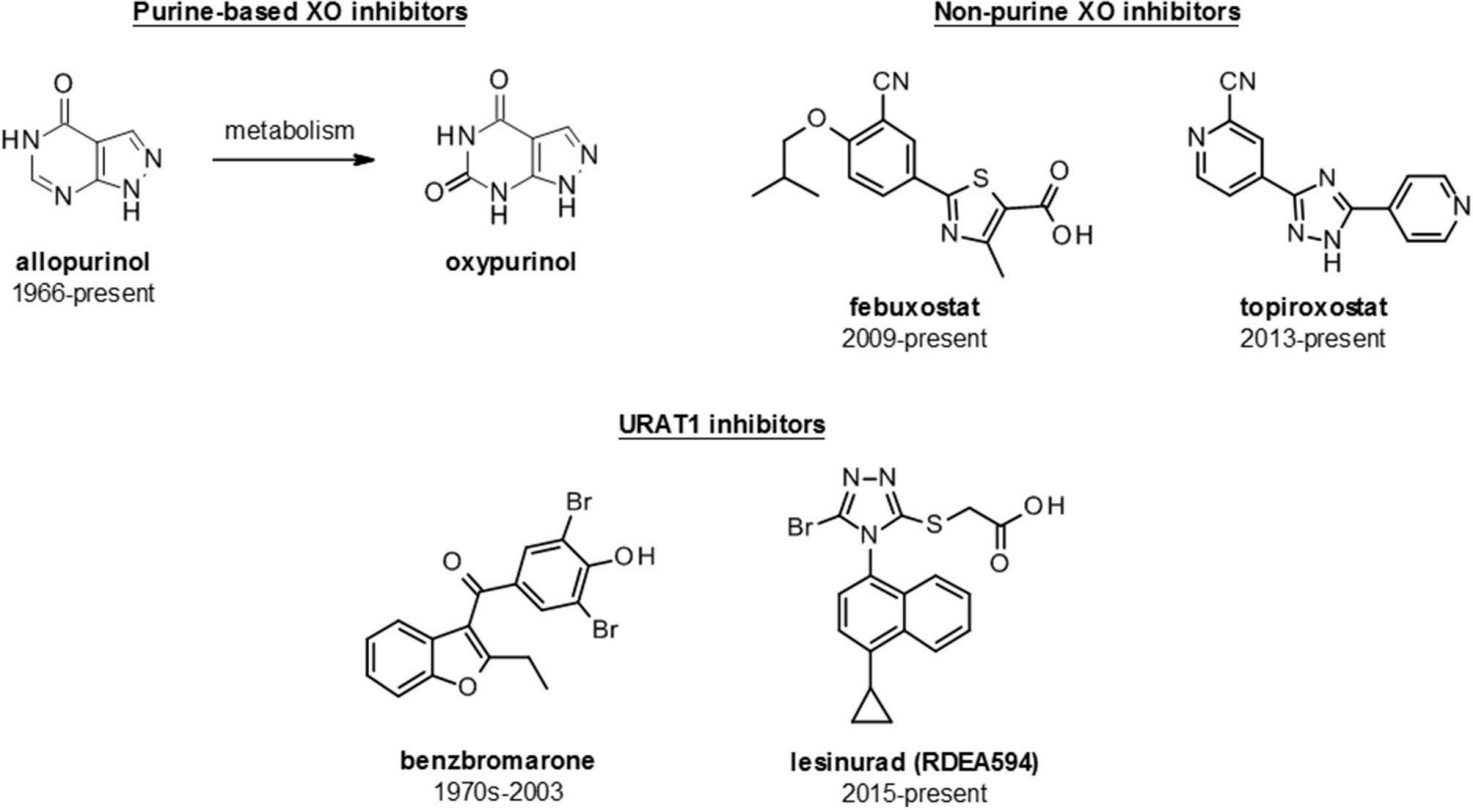
Structures of launched (marketed and withdrawn) drugs for the management of hyperuricemia. Purine-based (allopurinol and oxypurinol) and non-purine based (febuxostat and topiroxostat) XO inhibitors are shown with dates of approval for clinical use. URAT1 inhibitors are represented by benzbromarone (withdrawn in 2003) and lesinurad (RDEA594).

There are several approved ULT drugs which fall into three main classes: reduction of uric acid synthesis (xanthine oxidase inhibitors); increasing uric acid excretion (uricosurics, e.g., URAT1 inhibitors); and enabling systemic metabolic hydrolysis of uric acid (urolytics, e.g., recombinant uricases) (Figure 5, Table 2).

**Table 2:** Launched (marketed and withdrawn) therapies for hyperuricemia/gout.

**Compound**	**Mode of action**	**Company**	**Specific indications/comments**	**FDA highlights of prescribing information (where available)**
Allopurinol	XO	Generic		
Febuxostat	XO	Takeda	Chronic management of hyperuricemia in patients with gout. Not recommended for the treatment of asymptomatic hyperuricemia	https://www.accessdata.fda.gov/drugsatfda_docs/label/2012/021856s006lbl.pdf
Topiroxostat	XO	Sanwa Kagake Kenkyusho and Fuji Yakuhin		
Benzbromarone	URAT1	Sanofi-Aventis		
Lesinurad	URAT1	AstraZeneca	Use in combination with a XOi for the treatment of hyperuricemia associated with gout in patients who have not achieved target serum uric acid levels with a xanthine oxidase inhibitor alone. Not recommended for the treatment of asymptomatic hyperuricemia. Should not be used as monotherapy	https://www.accessdata.fda.gov/drugsatfda_docs/label/2015/207988lbl.pdf
Pegloticase	Uricase	Savient Pharmaceuticals	Treatment of chronic gout in adult patients refractory to conventional therapy	https://www.accessdata.fda.gov/drugsatfda_docs/label/2012/125293s034lbl.pdf
Rasburicase	Uricase	Sanofi-Aventis	Initial management of plasma uric acid levels in pediatric and adult patients with leukemia, lymphoma, and solid tumor malignancies who are receiving anti-cancer therapy expected to result in tumor lysis and subsequent elevation of plasma uric acid	https://www.accessdata.fda.gov/drugsatfda_docs/label/2009/103946s5083lbl.pdf

*XO(i), xanthine oxidase (inhibitor)*.

### Xanthine oxidase inhibitors (XOi)

The inhibition of xanthine oxidase reduces endogenous production of uric acid and thus lowers sUA levels. Xanthine oxidase inhibitors, in the form of allopurinol, were the first class of urate lowering therapy to reach the market and remain the first line therapy for hyperuricemia and gout. Inhibitors fall into two main classes: the classical purine analogs (including allopurinol) and more recently developed non-purine analog compounds such as febuxostat and topiroxostat.

#### Allopurinol

Allopurinol, a structural isomer of hypoxanthine, has been the cornerstone of hyperuricemia and gout clinical management since its introduction in 1966; and indeed remains the current standard of care despite some tolerability issues and reportedly low patient compliance (reviewed in (219)). It should be noted that patient adherence issues may simply reflect the high prevalence of allopurinol use over other urate lowering therapies, rather than reflecting any specific property of allopurinol.

Allopurinol itself is a relatively weak competitive XO inhibitor and is rapidly metabolized to the more potent oxypurinol, an isostere of xanthine, which is then renally cleared. Clinically, allopurinol is used for reducing uric acid levels particularly in the context of gouty arthritis and kidney stones/lithiasis. Additional indications include genetically linked enzyme disorders associated with uric acid overproduction, such as Lesch-Nyhan syndrome (HGPT deficiency), and myeloproliferative disease (tumor lysis syndrome). Allopurinol has been proposed to possess additional pharmacology, such as decreasing blood pressure and creatinine levels, which supports the notion that XO inhibition may have effects independent of urate lowering (220) and reviewed further in Richette et al. (150).

Although allopurinol remains the most frequently prescribed ULT, studies have suggested that less than 50% of patients taking the drug achieve a sUA level <6 mg/dL at an allopurinol dose of 300 mg/day (221, 222) and reviewed further in (138). A more recent study by Jennings suggests that of 400 patients, 36% required allopurinol up-titration to achieve sUA levels of <6 mg/dL (223). The recommended starting dose of allopurinol in the USA is 100 mg/day with incremental dose increases each 2–4 weeks, up to 800 mg/day^1^ (900 mg/day in Europe 224)), until the target of sUA <6 mg/dL is achieved (134, 135). However, in routine clinical practice, patients are often started at 100 mg daily and titrated up to 300 mg daily only, with the result that studies report between 36 and 50% of patients fail to achieve target sUA levels of less than 6 mg/dL (223, 225) and discussed further in Shahid and Singh (226).

Allopurinol has been associated with several adverse effects including gastrointestinal effects, rash and Stevens-Johnson's syndrome (227). In addition, allopurinol hypersensitivity syndrome (AHS) is a rare but potentially lethal risk for 2–8% of patients (228). Complications can also arise when patients have renal impairments which may require dose reductions (229). Some drug interactions with ampicillin or amoxicillin are known to increase the incidence of skin rash when used in combination (230). More recently, structural understanding of XO together with rational drug development has enabled the discovery of novel, chemically diverse and more potent XO inhibitors such as febuxostat.

#### Febuxostat

Febuxostat is a selective, non-purine, inhibitor of XO approved by the FDA in 2009, for management of patients with hyperuricemia in patients with gout but not for asymptomatic hyperuricemia^2^ Febuxostat is approved for use at doses of 40 and 80 mg/day in the USA; and up to 120 mg/day in Europe and 10–60 mg/day in Japan (18). Febuxostat has a reported IC_50_ of 1.8 nM, which is significantly more potent than allopurinol (IC_50_ 7.8 μM), and consequently has been shown to be more efficacious at doses of 80 or 120 mg for achieving target urate levels of <6 mg/dL vs. allopurinol at 100–300 mg daily (231). However, at the doses licensed for use in the USA, only 48–67% of those receiving febuxostat reach sUA levels <6 mg/dL (232).

Febuxostat clearance is predominantly via hepatic metabolism which suggests the potential at least for broader prescribing with respect to impaired renal function though this has not been fully assessed in the clinic. In addition, febuxostat has been reported to have fewer drug-drug interactions than allopurinol and is better tolerated in patients with AHS (233). It also has been reported to be a strong ABCG2 inhibitor, though the potential impact of this is not yet clear (234).

Interestingly, gout flares on commencement of treatment have been reported to be more frequent with febuxostat than with allopurinol, likely due to the more rapid and pronounced sUA reduction, an observation which has interesting implications with respect to considering the rate of sUA decrease for ULT strategies (235). While febuxostat is an option for patients with prior rash or hypersensitivity reaction to allopurinol, it is worth noting that a hypersensitivity reaction has also been reported with febuxostat (236). More recently, febuxostat was found to be noninferior to allopurinol with respect to rates of adverse cardiovascular events while all-cause mortality and cardiovascular mortality were reported to be higher with febuxostat than with allopurinol (237).

Clinical uptake of febuxostat has been relatively poor, likely in most part due to the cost compared to the generic allopurinol, with cost per tablet of $7 for febuxostat vs. $0.20–0.60 for allopurinol in the USA (226, 238, 239). In line with this, febuxostat has been suggested as second line to allopurinol in updated care guidelines (18).

#### Topiroxostat

Topiroxostat (FYX-051) is a structurally distinct, non-purine, selective XO inhibitor which was approved for use only in Japan in 2013 and was co-developed and marketed as Uriadec/Topiloric by Sanwa Kagake Kenkyusho and Fuji Yakuhin. It is available in oral tablets of 20, 40, and 60 mg doses and the general recommendation is to start with an initial 20 mg dose twice daily with the maximum approved dose being 80 mg twice daily with clinical efficacy reported at 120 mg/day (240, 241). Topiroxostat has been shown to inhibit xanthine oxidase via formation of a hydroxylated 2-pyridine metabolite that forms a covalent linkage to the molybdenum via oxygen and also interacts with amino acid residues in the solvent channel (242, 243).

### Uricosurics

Studies have suggested that, despite the standard of care for hyperuricemia and gout being focussed on reduction of synthesis by XO inhibitors, the underlying cause of hyperuricemia in a notable subset of patients is due to renal underexcretion of uric acid (110, 244). This suggests the application of uricosuric agents to increase renal urate excretion to be a rational approach for the treatment of hyperuricemia. However, by enhancing the renal clearance of uric acid, uricosurics may increase the risk of renal adverse events (e.g., nephrolithiasis) (reviewed in (245)).

Historically, several drugs, including probenecid, sulfinpyrazone, fenofibrate and losartan, were serendipitously discovered to have uricosuric properties although the underlying pharmacology was not initially understood (35, 139, 246). Likewise, benzbromarone, the first drug specifically developed for its uricosuric properties, was discovered and developed without any understanding of its pharmacology at a molecular level (reviewed in (247)). More recently, the identification of the URAT1 transporter and its role in urate re-uptake from the proximal tubule has shed light on the pharmacology of these compounds and enabled the development of a new generation of specifically targeted uricosuric compounds (39). Interestingly other transporters (e.g., GLUT9/*SLC2A9*) have also been genetically linked with urate homeostasis and may provide further targets for novel uricosuric drug development in the future (39, 59, 218).

Currently uricosurics are recommended as a second line therapy when target sUA levels are not reached, particularly as add-on therapies in combination with XO inhibitors (134, 135). Combination therapy with allopurinol and benzbromarone has been suggested to provide enhanced urate lowering and potentially more rapid resolution of tophi (145); a concept which has been applied to other XOi and uricosuric combinations.

#### Probenencid

Probenecid was originally introduced to prolong the action of antibiotics by reducing their renal clearance and functions as a non-selective inhibitor of organic anion transporters (reviewed in (248)). URAT1 has now been demonstrated to be one of the molecular targets of probenecid and, while its lack of selectivity and subsequent potential for drug-drug interactions limit its clinical use as a uricosuric, it perhaps represents the prototypical URAT1 inhibitor and uricosuric mechanism for ULT (92, 249).

#### Benzbromarone

Benzbromarone, first marketed in the 1970s, was the first compound specifically introduced as a uricosuric agent. The molecular basis of its pharmacology was not understood at the time of its introduction but it has subsequently been shown to be a potent URAT1 inhibitor (IC_50_ 22 nM) (96). Benzbromarone is effective as a single agent ULT with studies showing that 92% (22/24 patients) of gout patients reached target levels of serum urate from a 200 mg/day dose (250). However, it should be noted that in a subsequent study, where dose was escalated for both benzbromarone and allopurinol in patients failing to achieve adequate control on the starting dose, both compounds performed equally (251).

Like allopurinol, benzbromarone required dose titration from 50 to 200 mg once daily to achieve maximum efficacy but unlike allopurinol, benzbromarone could be used in patients with renal impairment. Given that uricosuric drug usage increases urate renal excretion, caution was advocated by clinicians where there was history of renal calculi so strategies such as urinary alkalinization to solubilize the uric acid were employed (245, 252).

Benzbromarone was not approved in the USA and was withdrawn from the market in many other countries in 2003 following reported incidents of idiosyncratic hepatotoxicity (reviewed in (247)).

#### Lesinurad (RDEA594)

Lesinurad (Zurampic®) is the first novel uricosuric to reach the market since benzbromarone (94). The primary mechanism of action of lesinurad derives from inhibition of URAT1, although it is also reported to be an inhibitor of OAT4 which could also potentially contribute to its efficacy; however, it is reported to be selective over related transporters such as OAT1 and OAT3 which may avoid some of the drug-drug interaction liabilities of non-selective compounds such as probenecid (94, 253–255, 273).

Following its acquisition by AstraZeneca in 2012, Ardea continued to progress the clinical programme. Phase 2 clinical studies have shown lesinurad to provide additional efficacy as an add-on therapy in individuals with inadequate urate reduction based on XOi's alone (256–258). Therefore, lesinurad was subsequently evaluated in three pivotal phase 3 trials in combination with XOi's; in combination with allopurinol in adults with gout showing inadequate response to allopurinol alone (CLEAR 1 and 2) and in combination with febuxostat in adults with tophaceous gout (CRYSTAL) (258–260).

The CLEAR 1 study demonstrated an increase in the proportion of patients achieving a serum UA level of <6.0 mg/dl after 6 months (the primary end point) with 54.2 and 59.2% in the lesinurad 200 mg plus allopurinol and lesinurad 400 mg plus allopurinol groups respectively, compared to 27.9% in those taking allopurinol alone. This was more broadly reflected across the duration of the trial with serum UA levels being lower at all time points assessed in both groups taking lesinurad plus allopurinol as compared with allopurinol alone. Lesinurad was reported to be generally well tolerated with the AE profile of the 200 mg lesinurad plus allopurinol dose comparable to that of allopurinol alone (73.1 and 68.7% respectively), while that of 400 mg lesinurad plus allopurinol was slightly higher (77.6%). Given the uricosuric mechanism of action, renal safety was highlighted as a potential risk and higher incidence of renal-related treatment-emergent adverse events (TEAEs) were observed with lesinurad 400 mg plus allopurinol (10.0%) compared with the lesinurad 200 mg plus allopurinol and allopurinol-alone groups (3.5 and 4.0%, respectively). In the lesinurad 400 mg plus allopurinol group a serious renal-related TEAE (renal failure) was reported in 1 patient (0.5%). The most common renal-related TEAE was elevations in serum creatinine ≥1.5 × baseline which occurred at higher rates in the lesinurad plus allopurinol groups (6.0 and 15.9% in the 200 mg and 400-mg lesinurad doses respectively) vs. allopurinol alone (1%). The CLEAR 2 trial showed similar findings with 55.4 and 66.5% in the lesinurad 200 mg plus allopurinol and lesinurad 400 mg plus allopurinol groups respectively, compared to 23.3% in those taking allopurinol alone achieving the target serum UA level of <6.0 mg/dl after 6 months. Likewise, the profile of renal-related TEAEs was comparable with lesinurad 400 mg plus allopurinol showing a higher rate (15.0%) compared with the lesinurad 200 mg plus allopurinol and allopurinol-alone groups (4.9 and 5.9%, respectively), again was mainly related to elevations in blood creatinine. Similar renal effects were also noted in a monotherapy trial with a 400 mg dose of lesinurad (261).

The CRYSTAL study investigated the safety and efficacy of lesinurad, in combination with febuxostat, in a 12-month trial in patients with tophaceous gout. The primary endpoint serum UA target, of <5.0 mg/dl after 6 months, was achieved by significantly more patients with febuxostat plus the addition of lesinurad 400 mg (76.1%; *P* < 0.0001), compared with febuxostat alone (46.8%). The 200 mg dose of lesinurad did not show a significant effect on the primary endpoint (56.6%; *P* = 0.13), however, at all other time points assessed, significantly more patients in this group achieved the serum UA target. There was no significant difference in the proportion of patients with complete tophus resolution between groups. However, the lesinurad (200 and 400 mg) plus febuxostat groups showed a greater reduction in the total target tophi area (50.1 and 52.9%, respectively) compared to febuxostat alone (28.3%). Overall rates of TEAEs were comparable across the group, while renal-related TEAEs were slightly increased in the lesinurad 200 mg plus febuxostat group, and in the lesinurad 400 mg plus febuxostat group (8.5 and 10.1%, respectively) compared with febuxostat (5.5%), again largely reflecting an increased incidence of elevated serum creatinine levels with lesinurad. No patients in the lesinurad 200 mg plus febuxostat group had a renal-related serious adverse event, while two patients in the lesinurad 400 mg plus febuxostat group (renal failure acute; renal failure chronic) and one patient in the febuxostat group (renal failure acute) had renal-related serious TEAEs.

Ultimately, the 200 mg dose of lesinurad received FDA approval at the end of 2015 for use in combination with a xanthine oxidase inhibitor for the treatment of hyperuricemia associated with gout in patients who have not achieved target serum uric acid levels with a xanthine oxidase inhibitor alone. Its use is contraindicated in patients with severe renal impairment, kidney transplant recipients, patients on dialysis, in tumor lysis syndrome (TLS) or Lesch-Nyhan syndrome)^3^ For more information a number of focused reviews of this compound have recently been published providing a more in-depth description of its profile (262–264).

### Urate hydrolysis by uricases (pegloticase and rasburicase)

Humans, and some primate species, differ from other mammals by the lack of a functional uricase enzyme. Uricase degrades uric acid into the more water soluble allantoin which is then eliminated readily through the kidney. Therefore, therapies based on the intravenous administration of a functional recombinant uricase enzyme have been developed which enable an immediate and significant reduction of sUA levels. Pegloticase, a PEGylated recombinant uricase was approved in 2010 for limited-patient use in the treatment of chronic refractory gout in patients with tophaceous deformities and complications (265, 266). The National Institute of Health and Clinical Excellence supports the use of pegloticase as a third line treatment for tophaceous gout in patients where XO inhibitors and uricosurics are contraindicated^4^; echoed by EULAR and ACR (American College of Rheumatology) guidelines (18, 134, 135). It is administered in 8 mg biweekly intravenous doses for at least 6 months. Clinical trials demonstrated a 47% response rate over placebo (266). However, likely due to the very rapid urate reduction caused by administration of pegloticase, its application has been associated with significant prevalence of acute flares. In addition, although pegloticase has been used effectively in the clinic it is also prone to immunogenic infusion reactions with a significant portion of the patients receiving pegloticase developing antibodies within the first few months of treatment (266). In clinical studies, infusion reaction adverse events occurred in 26–41% of patients including dyspnea, chest discomfort and flushing. These reactions were more common in patients in which significant levels of antibodies to pegloticase had developed. In order to reduce these infusion reactions, pre-treatment of patients with antihistamines or corticosteroids is carried out with the risk of complications that can lead to issues in patients with congestive heart failure (266).

A non-PEGylated uricase, rasburicase has also been approved for specifically treating hyperuricemia associated with tumor lysis syndrome as its quick mode of action and short half-life relative to pegloticase (8 h vs. 12 days) aligns well with this particular application (267). As a result, it was approved for the initial management of plasma uric acid levels in pediatric and adult patients with leukemia, lymphoma, and solid tumor malignancies who are receiving anti-cancer therapy expected to result in tumor lysis and subsequent elevation of plasma uric acid. Rasburicase also causes high rates of immunogenic infusion reactions, methemoglobinemia, hemolysis and anaphylaxis in patients which limit its more general use.

## Emerging clinical treatment approaches

Several factors may have influenced what appears to be a renewed interest in the development of treatments for hyperuricemia and gout in recent years, including: (1) emerging clinical perspective that gout and hyperuricemia are eminently treatable but under-treated conditions; (2) a growing understanding of relevant urate transporters in the kidney as potential drug targets enabling drug design; (3) observations that support the notion that hyperuricemia might contribute to a range of co-morbidities including hypertension, cardiovascular disease, stroke, type 2 diabetes, obesity, metabolic syndrome hyperlipidemia and chronic kidney disease; (4) adoption in Japan of an aggressive approach to the management of asymptomatic hyperuricemia creating a specific market opportunity.

**Figure 6:**
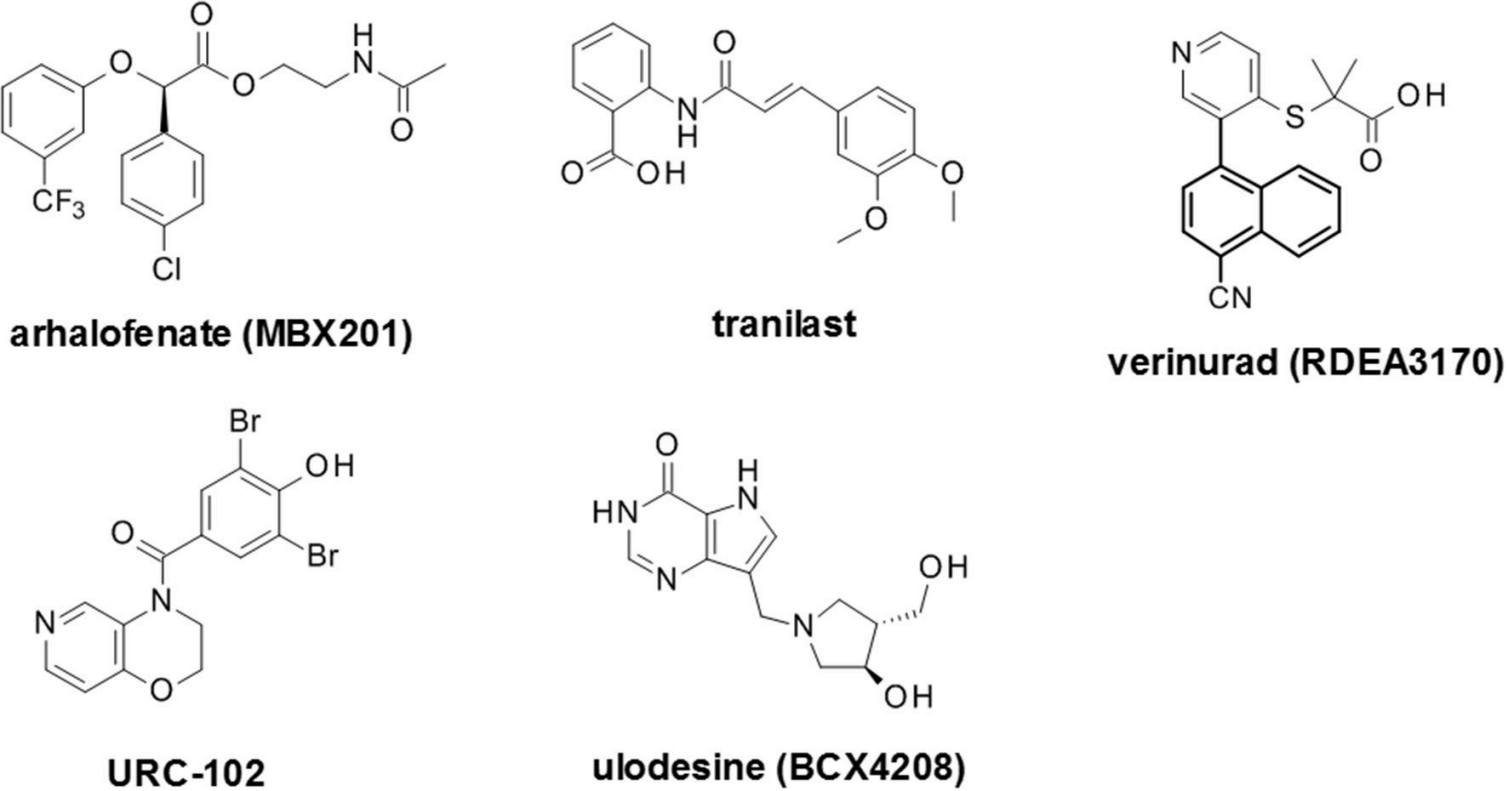
Selected recent emerging clinical compounds (where structure disclosed). The structures for arhalofenate (MBX201), tranilast, verinurad (RDEA3170), URC-102 and ulodesine (BCX4208) are shown here.

In this section, we have endeaveoured to identify and discuss candidates which are actively undergoing clinical assessment as either urate lowering therapies or for the control of gout flares (Figure 6, Table 3). Compounds which have not appeared to have advanced in clinical studies over the past 2 years, based on reported activity on Clintrials.gov, have been excluded as these are presumed to have been paused or stopped in clinical development although the rationale for this may not have been disclosed. A few notable examples of such compounds are briefly discussed at the end of the section based on scientific interest e.g., novel chemotypes for existing mechanisms, or a novel mechanism of action.

**Table 3:** compounds currently in development for hyperuricemia/gout.

**Compound**	**Alias**	**Target**	**Company**	**Website(s)**	**Phase**	**ClinTrials.gov last ongoing (recruiting)/completed activity related to hyperuricemia/gout**.	**References**
FYU-981		URAT1	Fuji Yakuhin, now co-developing with Mochida	http://www.mochida.co.jp/english/business/	3	Last study completed 2016, (NCT02416167, last updated February 2017)	(268, 269)
Arhalofenate	MBX201	interleukin-1β (IL-1β) modulator, reported to be a weak inhibitor of URAT1, OAT4 and OAT10	Cymabay licensed to Kowa	http://www.cymabay.com/pipeline.html, http://www.kowa.co.jp/eng/company/division/pharmaceutic/labo.htm	2b	Last study completed 2016, (NCT01399008, last updated September 2015)	(270–272)
URC-102		URAT1	Chugai/JW Pharmaceuticals	https://www.chugai-pharm.co.jp/english/ir/reports_downloads/pipeline.html http://www.cwp.co.kr/pharma/en/randd/develop.jsp	2	Last study completed 2015, (NCT02290210, last updated March, 2017)	
AC-201	Diacerein	interleukin-1β (IL-1β) modulator	TWi Biotechnology	http://www.twibiotech.com/rAndD_11	?	Last study completed 2016 (NCT02287818, last updated April 2017)	
Verinurad	RDEA-3170	URAT1i	AstraZeneca	https://www.astrazeneca.com/our-science/pipeline.html	2	Ongoing trial (NCT03118739—last updated July 2017)	(273)

*Inclusion in this table was based on evidence of trial activity on Clintrials.gov within the 2 years prior to compilation and listing on company websites. Other compounds may be in active development that do not meet this criteria*.

### Uricosuric compounds

#### Arhalofenate (MBX201)

Arhalofenate (Cymabay Therapeutics/Metabolex) is the (*R*) single enantiomer of the racemic drug halofenate that had been originally developed as a PPARγ inhibitor which was assessed in hyperlipidemia and diabetes but has since been repurposed as a gout drug based on urate lowering observations (274). Arhalofenate has been reported to act as a pro-drug which is cleaved *in vivo* to the active metabolite arhalofenic acid which has uricosuric activity attributable to the weak inhibition of a panel of renal uric acid transporters including URAT1, OAT4 and OAT10^5^ Interestingly, arhalofenate appears to have additional effects with respect to reducing the IL-1β signaling cascade in response to monosodium urate crystals, in addition to decreasing triglycerides and enabling insulin sensitization. This has been reported to lead to reduced incidence of flares in patients with gout in a similar manner to colchicine, which also inhibits the release of IL-1β (272, 275, 276).

The potential for arhalofenate (600 and 800 mg) to reduce flare rate was tested against allopurinol (300 mg) and allopurinol (300 mg) + colchicine (0.6 mg); the efficacy of the compound, as measured by percentage reduction in serum urate and the proportion of patients with a serum UA level of <6 mg/dL was included as a secondary outcome (277). Treatment with 800 mg arhalofenate led to a decreased number of gout flares with a 46 and 41% vs. allopurinol and placebo groups respectively. While allopurinol (300 mg) + colchicine (0.6 mg) showed a numerically greater reduction to arhalofenate 800 mg it was not statistically different. At week 12 the mean reduction in sUA level from baseline was −12.5% in the 600 mg arhalofenate group and −16.5% in the 800 mg arhalofenate group, compared with −0.9% in the placebo group. This compared to −28.8% in the 300 mg allopurinol group and −24.9% in the 300 mg allopurinol + 0.6 mg colchicine group.

The efficacy and tolerability of arhalofenate have been evaluated both as a monotherapy at doses of 600 and 800 mg once daily and in combination with febuxostat 40 and 80 mg (276). The primary objectives of the study were to assess reduction in sUA with arhalofenate as a monotherapy and when combined with febuxostat as both absolute and percentage reductions from baseline, the percentage of patients achieving the target sUA of <6, <5, and <4 mg/dL, and the potential for interaction between the two compounds. Arhalofenate as a monotherapy showed a modest reduction in sUA of approximately 19–24%. However, in combination with 40 or 80 mg febuxostat all patients were reported to achieve the target sUA of <6 mg/dL. Additionally, more subjects (0 vs. 20% and 29 vs. 79% for 40 and 80 mg of febuxostat vs. combination with 800 mg arhalofenate) achieved the more stringent goal of <4 mg/dL. Combination with febuxostat did not markedly affect the pharmacokinetics of arhalofenate. Overall these results were an improvement over either arhalofenate or febuxostat monotherapies which were monitored as comparators.

Arhalofenate was recently licensed to Kowa Pharmaceuticals^6^ suggesting the compound may progress to Phase 3 trials and, if successful, could become the first compound to combine urate-lowering and flare reduction in a single agent.

#### AC-201 (diacerein/diacetylrhein)

AC-201 (TWi biotechnology) is a small molecule IL-1β modulator that was originally developed for use in osteoarthritis. However, it has since been evaluated more broadly for metabolic indications including treatment of type 2 diabetes and gout flare prophylaxis. Indeed, AC-201 has been approved as diacerein for chronic rheumatic disease and is already in clinical use. However, clinical data suggested potential dual effects in reducing sUA levels through inhibition of reabsorption transporters in the kidney. This molecule is currently listed as being actively assessed in Phase 2 clinical development at 50 mg dosage (including a controlled release formulation). A trial was carried out to determine the capability to reduce flares and time of onset to flares for patients who started on urate lowering therapies^7^ with the proportion of patients meeting the goal of <6.0 or <5.0 mg/dL assessed over 16 weeks. Overall the possible dual action of reduction in flares combined with increases in urate excretion could prove beneficial relative to other emerging drugs in this area and represents a potential competitor to arhalofenate with a dual anti-inflammatory/sUA lowering profile. However, currently there are only limited data available for this compound in the public domain (226).

#### RDEA3170 (verinurad)

Further optimization of the RDEA594 (lesinurad) template culminated in the development of RDEA3170 (verinurad) which reportedly has a URAT1 IC_50_ of 25 nM and is currently in Phase 2 clinical trials (97, 255, 273). This is more potent than lesinurad which has been reported to have a URAT1 IC_50_ of 3.4 μM. Verinurad completed a Phase I clinical trial, in Japanese patients with gout or with asymptomatic hyperuricemia, which was designed to demonstrate its urate lowering effects (94, 278). Doses ranging from 5 to 12.5 mg per day were given and resulted in >60% decrease in the mean sUA levels in patients. Phase 2 trials of verinurad in combination with either febuxostat or allopurinol were then initiated (279, 280).

#### FYU-981

FYU-981 (Fuji Yakuhin Co.) is in Phase 3 clinical trials for patients with hyperuricemia both with and without gout^8^ However, despite being in advanced clinical trials, very little appears to have been published about this compound to date.

#### URC-102 (UR-1102)

URC-102 (JW Pharmaceutical) has recently been through Phase 2 clinical trials to assess efficacy and safety in gout patients at 0.25 mg to 2.0 mg doses^9^ Published studies on this compound showed selective inhibition of the URAT1 transporter (281). However, no clinical data for this compound has been reported.

### Selected compounds without recent clinical progress

In addition to the aforementioned compounds which appear to be still actively progressing in clinical trials, there are a number of recent compounds for which no recent clinical activity has been reported. These include alternative novel XO inhibitors such as LC350189 by LG Life Sciences for the management of hyperuricemia in gout patients (282). Also, alternative uricosuric compounds were progressed into clinical trials, such as the proposed re-purposing of tranilast, an anti-inflammatory drug which has been used for many decades in Japan for bronchial asthma that had a secondary uricosuric effect. This uricosuric effect was later attributed to weak inhibition of GLUT9 in addition to URAT1, OAT4 and OAT10 (283). In addition, tranilast appears to inhibit a number of secretory urate transporters including NPT1, OAT1 and OAT3 which may also contribute to this effect to some extent. A novel dual XO and URAT1 inhibitor, PF-06743649, combined both inhibition of uric acid biosynthesis and increase urate excretion and was evaluated in partnership by Kissei Pharmaceuticals and Pfizer up to Phase 2 clinical trials for hyperuricemia and gout treatment, but was terminated due to emergent renal safety risks (284). Ulodesine (BCX4208) is also noteworthy as it represented a novel mode of action, outside of XO inhibitors, uricosuric and uricolytic agents. This is a potent purine nucleoside phosphorylase (PNP) inhibitor acting upstream of xanthine oxidase inhibitors (285, 286).

## Conclusions

Asymptomatic hyperuricemia appears to be increasing in prevalence. High levels of circulating uric acid have been associated with the increased risk for developing gout and kidney stones. Indeed, gout is the most common form of inflammatory arthritis with well-studied epidemiology. The natural history of gout can be summarized as asymptomatic hyperuricemia, acute gouty arthritis, intercritical period and chronic tophaceous gout; with diagnosis based on both laboratory and radiological features. The role of genetic predisposition is becoming more evident and new insights into the pathophysiology of hyperuricemia and gouty arthritis (both acute and chronic) allow for an even better understanding of the disease.

Gout is an eminently curable condition with approaches that include management of the inflammatory pain associated with flares, urate lowering therapies that address the underlying cause, as well as other approaches such as improved patient education. It has been suggested that clear visualization of treatment outcome could, in combination with improved patient education, help address the issue of poor patient compliance (287). If the treatment paradigm shifts further toward active management of hyperuricemia, we anticipate that patients will benefit from more facile methods for determining uric acid concentrations in biofluids which, together with increased availability of mobile applications (such as smart-device applications), has the potential to allow non-invasive self-assessment and subsequent disease management.

The associations between hyperuricemia and many co-morbidities such as hypertension, cardiovascular disease, diabetes, metabolic syndrome, dyslipidemia, acute and chronic kidney disease are well recognized. However, the causal relationship between hyperuricemia and these co-morbidities is still a subject of debate. Recent years have witnessed many experimental, epidemiological (including Mendelian randomization studies) and clinical therapeutic interventional studies seeking to test the hypothesis that hyperuricemia plays a pathophysiological role in these disease entities, many of which are important public health challenges. These investigations have been limited by the lack of a standardized approach for definition of hyperuricemia, and by duration and/or size. Nonetheless, the results are interesting enough to warrant further investigation with larger randomized controlled trials to establish the role of uric acid as a promising target for novel therapeutic interventions in the management of kidney and cardio-metabolic disease. Indeed, there have been notable efforts to standardize medical approaches regarding outcome measures, staging and management but there are still some notable gaps.

For example, despite ongoing efforts to educate practitioners on the appropriate titration of allopurinol (which is still often used as the therapy of choice), prescribed doses are typically around 300 mg in clinical practice which in turn leads to suboptimal outcomes. However, the increasing number of therapeutic options may help address this issue by removing requirements for dose titration, as is the case with newer XO inhibitors, or using combinations of XO inhibitors and uricosurics in patients who do not achieve sUA goals with XO inhibitors alone. Finally, further agents in development will likely benefit from our better understanding of pathophysiology. Taken together, there is genuine potential for further drug development to lead to better management in patients with hyperuricemia, gout and associated conditions in the coming years.

## Author contributions

CB conceived the manuscript. CB, RS, AP, CV, PL and RG collected and reviewed the literature and wrote the main body of the manuscript. PL and PD critically reviewed the manuscript. All authors approved the final version of the manuscript.

### Conflict of interest statement

CB is a paid employee of Astex Pharmaceuticals. PD, RG, and PL are paid employees of Pfizer Ltd. RS and AP are paid employees of AstraZeneca and inventors on patents for URAT1 inhibitors (not in active clinical development at the time of submission). CV is a paid employee of Genomics plc.

## Footnotes

^1^https://www.accessdata.fda.gov/drugsatfda_docs/label/2017/209203s000lbl.pdf

^2^https://www.accessdata.fda.gov/drugsatfda_docs/label/2012/021856s006lbl.pdf

^3^https://www.accessdata.fda.gov/drugsatfda_docs/label/2015/207988lbl.pdf

^4^https://www.nice.org.uk/guidance/ta291

^5^http://ard.bmj.com/content/annrheumdis/71/Suppl_3/450.3.full.pdf

^6^http://www.kowapharma.com/newsroom/pr-2017-01-03/

^7^https://clinicaltrials.gov/ct2/show/NCT02287818

^8^https://clinicaltrials.gov/ct2/show/NCT03006445

^9^https://clinicaltrials.gov/ct2/show/NCT02290210
